# Steered Molecular Dynamics of Lipid Membrane Indentation by Carbon and Silicon-Carbide Nanotubes—The Impact of Indenting Angle Uncertainty

**DOI:** 10.3390/s21217011

**Published:** 2021-10-22

**Authors:** Przemysław Raczyński, Krzysztof Górny, Piotr Bełdowski, Steven Yuvan, Beata Marciniak, Zbigniew Dendzik

**Affiliations:** 1 Faculty of Science and Technology, University of Silesia in Katowice, 41-500 Chorzow, Poland; przemyslaw.raczynski@us.edu.pl (P.R.); krzysztof.gorny@us.edu.pl (K.G.); 2 Department of Chemistry, Surface and Corrosion Science, School of Engineering Sciences in Chemistry, Biotechnology and Health, KTH Royal Institute of Technology, Drottning Kristinas Väg 51, SE-10044 Stockholm, Sweden; piotr.beldowski@pbs.edu.pl; 3 Institute of Mathematics and Physics, Bydgoszcz University of Science and Technology, 85-796 Bydgoszcz, Poland; 4 Department of Physics, East Carolina University, Greenville, NC 27858, USA; yuvan@ecu.edu; 5 Faculty of Telecommunications, Computer Science and Electrical Engineering, UTP University of Science and Technology, 85-796 Bydgoszcz, Poland; beata.marciniak@pbs.edu.pl

**Keywords:** nanotubes, steered molecular dynamics, biological membranes, indentation, drug delivery, nanoneedle

## Abstract

Due to the semi-liquid nature and uneven morphologies of biological membranes, indentation may occur in a range of non-ideal conditions. These conditions are relatively unstudied and may alter the physical characteristics of the process. One of the basic challenges in the construction of nanoindenters is to appropriately align the nanotube tip and approach the membrane at a perpendicular angle. To investigate the impact of deviations from this ideal, we performed non-equilibrium steered molecular dynamics simulations of the indentation of phospholipid membranes by homogeneous CNT and non-homogeneous SiCNT indenters. We used various angles, rates, and modes of indentation, and the withdrawal of the relative indenter out of the membrane in corresponding conditions was simulated.

## 1. Introduction

While commercial adoption of carbon nanotubes (CNT) has stumbled over problems of cost and scale, its use in the sciences has flourished. Research into carbon nanotubes began its impressive expansion at the start of the century [1], driven by a likewise impressive track record of progress and perhaps public enthusiasm. CNTs and other carbon-based nanostructures have found meaningful applications that take advantage of nearly every individual and bulk property they possess—as conductors, as semiconductors, and as thermal transport due to their physical rigidity, flexibility, and chemistry [2]. Biomedical advancements have been equally numerous [3,4,5,6], owed primarily to the ability of CNTs to maintain their attractive physical properties while also being highly compatible with organic molecules. In the field of tissue engineering, for example, CNTs’ similarity in scale to extracellular proteins opens the door for influencing cell growth and organization or enhancing the physical properties of agarose media [7]. This potential was demonstrated in [8], where polyethylene glycol single-walled carbon nanotubes (PEGSWCNTs) combined with gene inhibitors were able to safely enter and influence chondrocytes after direct injection into the synovial cavity of mice.

One of the appealing properties of CNTs is their ability to be functionalized and adhere to biologically active molecules. This proved important for reducing toxicity [5,9], but also greatly expands potential applications of CNTs. Combined with their electrochemical and infrared luminescent properties, it has led to successful construction of nanotube segments that sense a range of physiological agents [10]. These types of sensors still rely, however, on cellular uptake mechanisms which increase the challenges in their design [11]. Indenters provide a more direct route to study cell interiors. As early as 2007 it was demonstrated that multiwalled CNTs affixed to atomic force microscopes could be used to penetrate membranes of individual cells or bacteria [12,13]. Biosensors must exhibit good physical and chemical properties and be biologically compatible. Due to their properties, CNTs can easily cross biological membranes, making them applicable in vivo with minimal invasiveness and further implemented to photoacoustics. CNTs can be made biocompatible through various dispersion and functionalization methods. As an example, the poly(ethylene glycol)-coated CNTs have also been used in the vectorization of anticancer agents, such as TRAIL (tumor necrosis factor-related apoptosis-inducing ligand), with a very high degree of success [14]. Additionally, protamine–CNT complexation could lead to increased biocompatibility of CNTs and enhance their ability to enter cells [15,16,17].

CNTs offer various features of interest to engineers to be useful as biosensors: they have a large specific surface area that enables the immobilization of many functional units such as receptor moieties for biosensing. Additionally, CNTs exhibit unique intrinsic optical properties such as photoluminescence in the near-infrared (NIR) and strong resonance Raman scattering, making them an excellent candidate for biological detection. Another attractive property is a photothermal response that could reduce the tumor size or be removed entirely by using NIR laser irradiation to generate heat. Different types of CNTs have different properties. SWCNTs, for example, can conduct electricity approximately 100 times better than copper, which can help target particular molecules. Numerous CNT biosensors have been established to date to examine an extensive range of cancer biomarkers through conjugation of DNA and aptamers, antibodies, peptides, proteins, or enzymes [18].

In this study, we continued to investigate the mechanical effects of single-walled CNTs penetrating and withdrawing from lipid membranes by extending simulations to non-idealized circumstances. As molecular dynamics simulations have showed mainly the idealized case when the angle is normal to the membrane’s surface, our goal was to establish how the uncertainty of angle from the ideal (normal) position influences the process of indentation. Understanding the process can help to design CNT-based nanosensors that can consider such uncertainty while designing and functioning. Prior work has assumed the nanotubes to be perfectly aligned both to the bilayer surface and the direction of indentation [19,20]. However, even in the context of atomic force microscopy, it can be challenging to achieve such conditions [21]. Furthermore, while imaging tips can be shortened to limit buckling [22], significant length and minimal diameters are desirable for probing beyond cell membranes and minimizing physical disturbance [23]. Here we characterize the force and membrane disruption over a range of conditions, including angle, speed, and indentation direction.

## 2. Simulations’ Details

**Figure 1:**
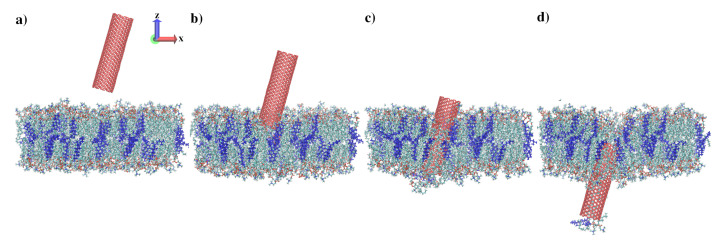
A series of snapshots visualizing tube-axis membrane indentation for CNT, 15°, and v=2.0 m/s. Cholesterol molecules inside the DMPC membrane are dark blue. (**a**) CNT initial stage (after equilibration); CNT indenter tip is at: (**b**) z=−25 Å, (**c**) z=−55 Å, (**d**) z=−85 Å.

We performed constant velocity steered molecular dynamics simulations [24,25] for indentation of a lipid membrane by carbon (CNT) and silicon carbide (SiCNT) nanotubes, and simulated their subsequent withdrawal. In this scheme, imaginary “steering” particles are attached by virtual springs with spring constant k = 10 $\;\mathrm{kcal}\;{\mathrm{mol}}^{-1}\;{Å}^{-2}$ to each of the atoms in the rearmost ring of the nanotube. After initial equilibration, the nanotube is then set in motion by giving these particles a specified constant velocity. Analogous (10, 10) armchair nanotube configurations where chosen for the CNT and the SiCNT. The approximate lengths and diameters for this configuration were 50 by 13 Å for the CNT and 72 by 16 Å for the SiCNT; the increased size of SiCNT is due to the greater length of C–Si bonds (1.78 vs. 1.42 Å). Not only may the indentation angle affect the characteristics of the process; the indenter length/aspect ratio has also been discussed in this context [26,27]. It has been predicted that reducing nanotube length may lead to more effective penetration of the membrane [26]. Models of phospholipid bilayers [28,29] and carbon and silicon carbide nanotubes have been described elsewhere [20,30,31]. The membrane consists of 232 1,2-dimyristoyl-snglycero- 3-phosphocholines (DMPC) and 48 cholesterol molecules. In the case of SiCNT, the system was solvated in approximately 39,000 water molecules; for CNT it was approximately 28,000 water molecules. Three indentation angles, 4.5°, 9°, and 15°, were chosen for the CNT, and only the most extreme angle, 15°, for the SiCNTs. All angles were relative to the membrane’s normal axis (Figure 1a). Each angle was simulated for two different modes: indentation directed along the nanotube axis (“tube-axis” indentation) and indentation directed perpendicularly to the membrane surface (“*z*-axis” indentation)—and for two speeds: v=0.5 m/s (low rate) and v=2.0 m/s (high rate). All simulations were performed at physiological temperature maintained by Langevin thermostat (damping coefficient $\gamma =1\;{\mathrm{ps}}^{-1}$) in an aqueous environment with a standard TIP3P water model [32]. NAMD version 2.8 [24] with a standard NAMD integrator (Brünger–Brooks–Karplus algorithm) [33] was used. To ensure sufficient energy conservation, the integration time step was equal to 0.5 fs. Periodic boundary conditions were applied for all systems examined. The size of the box, after equilibration, was approximately equal to 102 × 82 × 137 Å for the systems with a CNT and 100 × 82 × 170 Å for the systems with a SiCNT. The interactions in the membrane were modeled with an all atom–atom CHARMM36 [34,35] force field and visualized with VMD software [36].

The initial configurations of all systems were obtained from a series of NPT simulations (2 × 10^6^ steps) with the pressure set to 1 atm controlled using Langevin barostat [37] implemented in NAMD, with piston fluctuation control implemented using Langevin dynamics as in [38]. During next phase of equilibration (2 × 10^6^ steps), and during the main simulations, a constant number of particles and constant volume were maintained (NVT ensemble). The duration of each simulation depended on the speed of the indenter and continued until the membrane was fully penetrated to properly assess the number of lipids removed from the bilayer. Subsequent withdrawal processes began from a snapshot of the system of interest taken when the tip of the indenter had reached the lower surface of the membrane.

Each process was repeated 15 times, and the results represent the data averaged over multiple trajectories. For the withdrawal process, four simulation states were taken as starting configurations from all trajectories with 15° angles. These corresponded to the two states with the closest to average numbers of lipids removed from the membrane and the two states with the maximum lipid removal. Each withdrawal was then simulated five times, and the results, for a total of 160 independent runs (4 cases × 4 configurations × 5 simulations = 80 runs for CNT and SiCNT each).

## 3. Results and Discussion

### 3.1. Indentation Process

Figure 1 shows four instantaneous snapshots for CNT-axis indentation with an angle of 15 degrees. The first snapshot (Figure 1a) shows the initial configuration. Figure 1b,c show the nanotube reaching the upper and lower surfaces of the membrane, as defined by the glycerol backbones of the constituent phospholipids. Following indentation, in most cases, a few lipid molecules were removed or found within the nanotube (Figure 1d).

**Figure 2:**
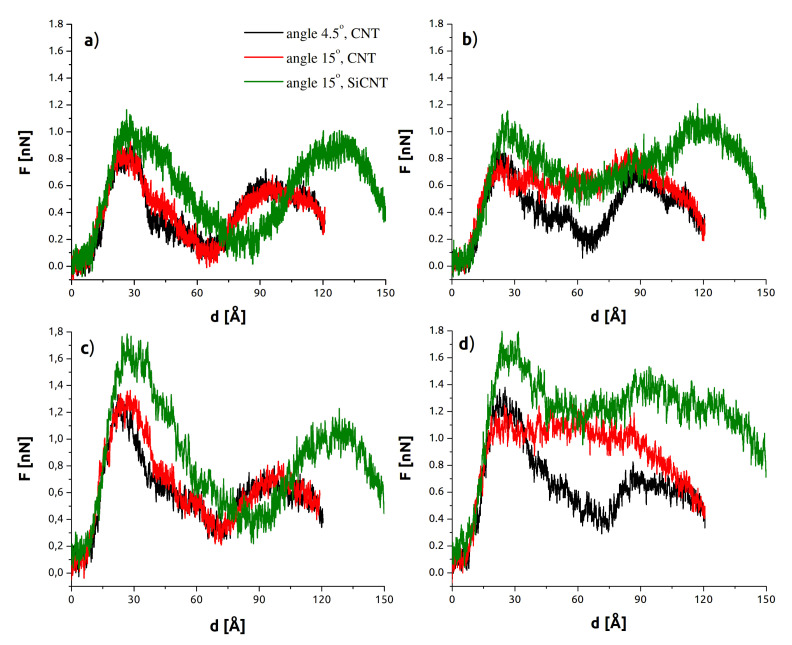
SMD force vs. indenter depth. (**a**) Low rate (v=0.5 m/s), tube-axis indentation. (**b**) Low rate (v=0.5 m/s), *z*-axis indentation. (**c**) High rate (v=2.0 m/s), tube-axis indentation. (**d**) High rate (v=2.0 m/s), *z*-axis indentation.

Figure 2 compares the average SMD forces for each set of parameters versus the depth, *d*. Note that *d* represents the *z*-axis measured depth of penetration into the membrane and does *not* equate to the distance of travel (which is greater for the tube-axis cases). Data for 9° tilt were similar to that for 4.5° tilt, and have been omitted for clarity (force data for 9° cases, and work plots and other omitted miscellany, have been included in the supporting information). The main comparison shown is between the two CNT curves in black (4.5°) and red (15°). The top row shows data for the lower rate of v=0.5 m/s, and the corresponding v=2.0 m/s cases are below. The overall force was higher for the higher rate (by about 0.2–0.5 nN), as one may expect. The first peak is associated with the upper (at approx. d=25 Å) membrane and the energy necessary to separate the lipid heads. The second peak (near d=100 Å) is much less sharp and represents an indenter position already substantially outside the membrane, similarly to Figure 1d. The energetically favorable configuration is for the nanotube to be immersed in the hydrophobic membrane’s interior. This second peak therefore did not arise from a discrete event but at the point these hydrophobic interactions were most rapidly broken. The values of SMD forces are in good agreement with those reported in the case of perpendicular indentation [12,30,39,40,41]. This suggests that in the case of limited deviation from idealized circumstances, the force required to successfully penetrate the membrane is not considerably higher than in the case of perpendicular indentation. The calculated interaction energies are presented in the Supplementary Material (Figure S2). The right half of Figure 2 contains data for *z*-axis indentation. In addition to the force increasing with angle, both the CNT and SiCNT 15° curves feature a plateau between peaks. This relationship is not prominent for indentation along the nanotube axis (Figure 2, left side), and there is no plateau. The conclusion is that what we refer to as *z*-axis indentation is more akin to cutting or slicing than the needle-like piercing of the other cases. Concomitant with this is a resistance to motion that must be continuously overcome as the nanotube disrupts additional lipid molecules during its motion. As a result of these increased viscous forces, the total SMD work required to penetrate the membrane is larger for *z*-axis cases than for their corresponding tube-axis cases (see supporting information).

In analogous models, SiCNTs about 20% larger than CNTs. This leads to larger forces overall, as shown in Figure 2. However, the characteristic plateau discussed in the last paragraph is considerably reduced in the *z*-axis plot. Following the peak at d=30 Å, the force does not level off until about d=50 Å. This is representative of the increased interaction of the SiCNT with the membrane, which exaggerates both the initial peak and the subsequent compensation just described. The stronger interaction and greater length of SiCNT are also visible in the dramatic rightward shift of the final force peaks throughout Figure 2 and the bending it induces in the membrane, discussed at the end of this section.

**Figure 3:**
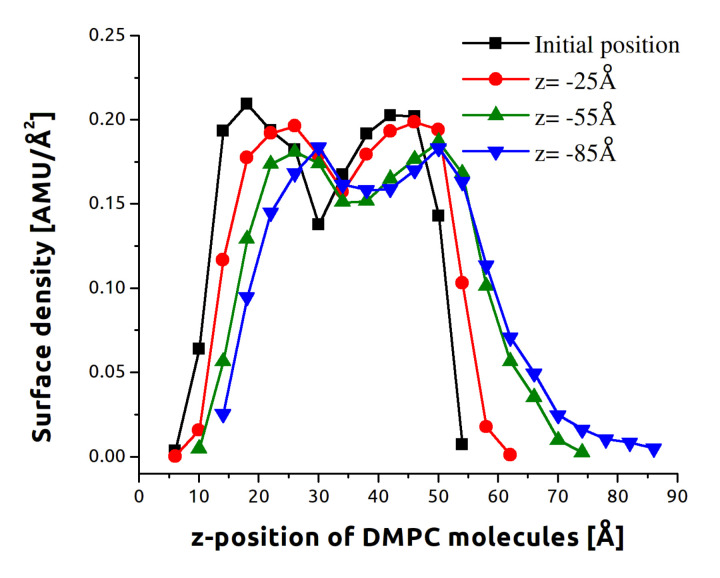
The average DMPC mass density profile along the *z*-axis. Data are for the SiCNT indenter at 2 m/s, a 15° angle *z*-axis indentation. Each point represents a 4 Å wide average across all equivalent trajectories for the instant specified in the inset.

Figure 3 demonstrates the impact to the membrane structure by SiCNT. The initial black curve represents the original density profile of the membrane. The two distinct peaks each represent one of the membrane surfaces. By the time the nanotube has exited, the border between the layers has been washed out and the surfaces are no longer distinct.

Table 1 quantifies the relative disruption of the membrane by the average (standard deviation) of lipids removed for each case. SiCNT removed the most lipids on average and the highest number of lipids in a single trajectory (19 lipids) during one *z*-axis indentation run. In all cases, indenters at greater angles removed more lipids and in all but a single case (CNT at 15° along the *z*-axis), faster indentation resulted in membrane sparing.

**Figure 4:**
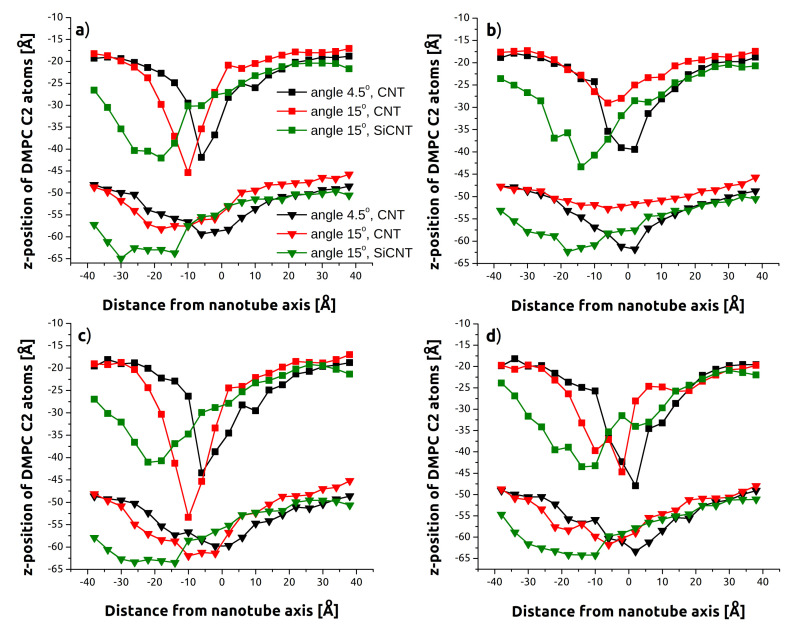
Average DMPC backbone C2 atom position at an indentation depth of z=−55 to a resolution of 4 Å. Both the upper and lower layers are shown. (**a**) Low rate (v=0.5 m/s), tube-axis indentation. (**b**) Low rate (v=0.5 m/s), *z*-axis indentation. (**c**) High rate (v=2.0 m/s), tube-axis indentation. (**d**) High rate (v=2.0 m/s), *z*-axis indentation.

Some of these results can be understood from Figure 4, which shows the displacement of backbone C2 atoms across a slice of the membrane containing the angled indenter. These deflection profiles correspond to the position in Figure 1c (z=−55 Å) where the nanotube tip is amidst the headgroups of the lower surface. Both the upper and lower layers are shown. On average, C2 atoms near the tip of the nanotube approach or exceed z=−45 Å–nearly reaching the bottom surface of the membrane—and demonstrating the strong tendency for the indenter to take some lipids along. It should be noted that even at this early stage of penetration for the lower layer, the much more dramatic impact of the greater surface area and interaction of SiCNT with the membrane are already visible. Viewing both the upper and lower layers together, the aforementioned membrane bending is clearly visible in contrast to the more localized punctures for CNT.

The locations of minima in Figure 4 are shifted to the left as a consequence of the lateral displacement of the nanotube tips, as the positive direction on the horizontal is the direction of tilt. This becomes most pronounced for tube-axis indentation as these indenters continue to travel laterally beyond their initial orientations. Apparent as well is the increased disruption caused by the “cutting” style penetration of *z*-axis indentation. These profiles skew noticeably to the right as the trailing length of the nanotube continues to push through the membrane and interferes with individual lipids returning to the upper layer. These effects are again larger for the SiCNT due to its greater size.

**Figure 5:**
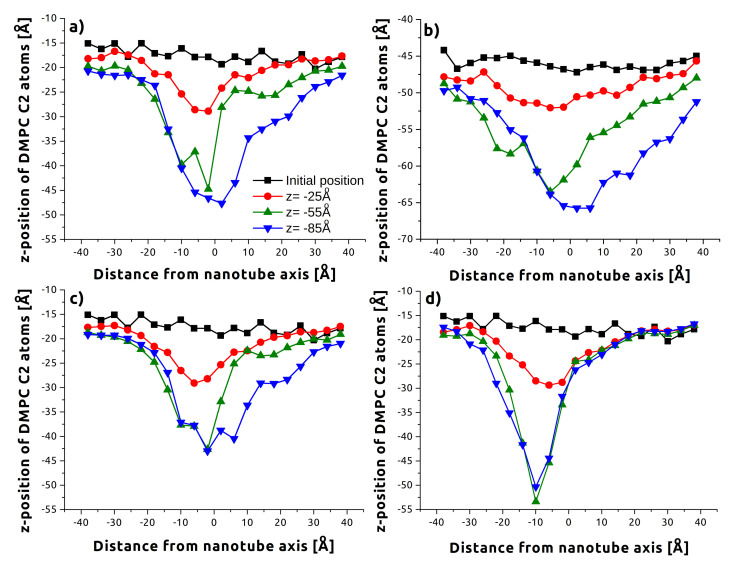
Sequential profiles of average DPMC backbone C2 atom position for CNT systems angled at 15° to a resolution of 4 Å. *Z*-values correspond to the stages shown in Figure 1. Each plot represents a different case: (**a**) *z*-axis indentation at 2 m/s—upper layer, (**b**) *z*-axis indentation at 2 m/s—lower layer, (**c**) *z*-axis indentation at 0.5 m/s—upper layer, (**d**) tube-axis indentation at 2 m/s—upper layer.

Despite the increased removal of lipids noted above, lower indentation rates (Figure 4 top half) caused less membrane deflection. This was true for both the upper and lower layers, but is more obvious for the upper layer in the data shown. This difference is examined more closely in Figure 5, which compares C2 atom deflection for 15° indentation at the successive instances of Figure 1: before contact, penetrating the upper layer of headgroups, penetrating the lower layer of headgroups, and exiting the membrane. The higher rate (Figure 5a vs. Figure 5c) has both greater displacement of lipids at the minima (47 Å vs. 43 Å) and the same across the membrane. This could be associated with the greater time the membrane has to accommodate the nanotube at slower speeds.

A sample lower membrane sequence is also shown in Figure 5b. It is worth mentioning that the deflections for the lower membrane could be due to two separate effects, from the nanotube itself but also due to lipid molecules that have been separated from the upper layer and pushed in front of the nanotube.

### 3.2. Withdrawal Process

**Figure 6:**
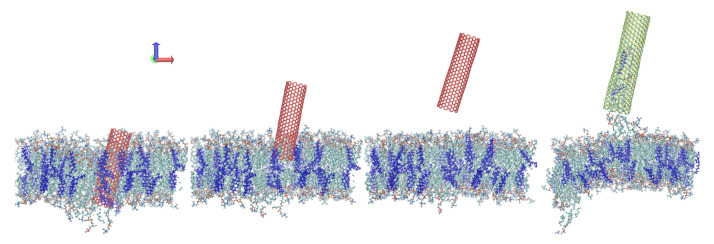
A series of snapshots visualizing tube-axis membrane withdrawal for CNT, 15°, and v=2.0 m/s. Cholesterol molecules inside the DMPC membrane are colored dark blue. (**a**) CNT indenter at z=−55 Å, (**b**) z=−25 Å, (**c**) z=5 Å; (**d**) SiCNT indenter at z=5 Å.

We applied the same methods of analysis to the withdrawal process. Figure 6 shows the stages of withdrawal process, and a final state for SiCNT showing the extent of lipid removal which can occur. Both modes (tube-axis and *z*-axis) and speeds were simulated, but only configurations from 15° tube angles were chosen, as they represent the most invasive scenarios simulated.

**Figure 7:**
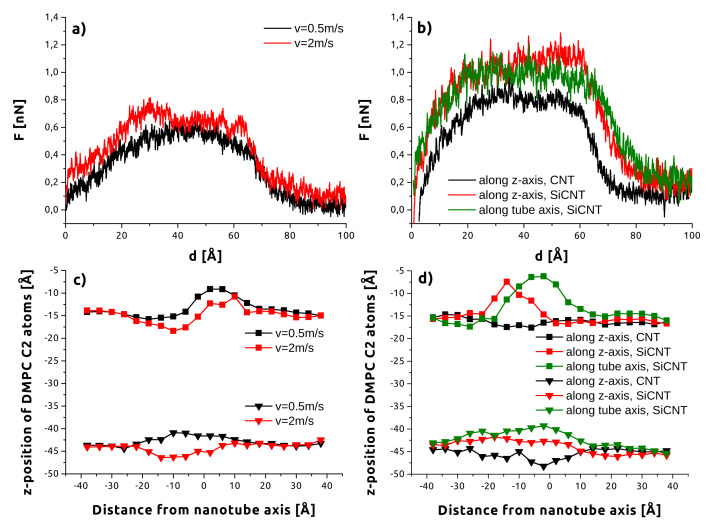
SMD forces vs. depth (top row) and backbone C2 atom positions along *x*-axis at indenter depth d=−25 Å (bottom): selected comparisons. All cases simulated were angled at 15°. For the C2 atom profiles, both membrane layers are shown. Panels (**a**,**c**): rate comparison (CNT, tube-axis indentation). Panels (**b**,**d**): nanotube comparison (CNT/SiCNT high rate indentation).

Figure 7 presents the most significant results and compares the force profile and membrane deflection for SiCNT and CNT. As the nanotubes do not need to re-penetrate the upper layer, the process is smoother overall. The efficiency with which the membrane self seals following CNT is visible in the rapid falloff in the force characteristic past 60 Å (Figure 7a). The deflection profile in Figure 7c shows that the membrane returns to normal by the time CNT reaches z=5 Å (e.g., Figure 6c), while significant deformation continues for SiCNT.

This difference is reflected in the tendency for SiCNT to permanently remove more lipids (Table 2). The difference is slight for a fast withdrawal but quite large for slow withdrawal. This suggests the hydrophobic “stickiness” of a SiCNT can be easily overcome by additional viscous force. For the lower rate, the disruption can be fairly dramatic, as in (Figure 6d), which shows not only lipids removed from the upper and lower surfaces, but a bending of the entire membrane similar to that described for indentation.

**Figure 8:**
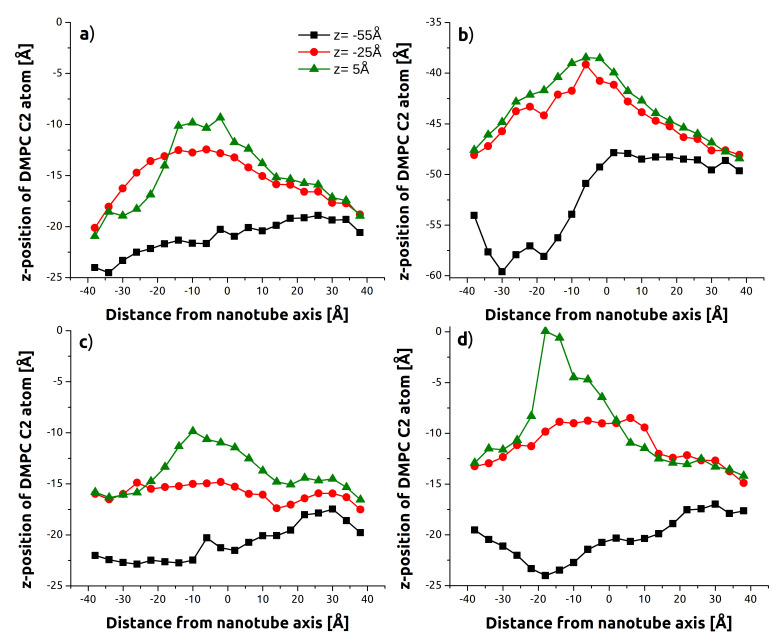
Sequential deflection profiles for SiCNT as illustrated by the average DMPC backbone C2 atom position to a resolution of 4 Å. (**a**) Low rate (v=0.5 m/s), tube-axis, upper layer; (**b**) Low rate (v=0.5 m/s), tube-axis, bottom layer; (**c**) High rate (v=2.0 m/s), tube-axis; (**d**) Low rate (v=0.5 m/s), *z*-axis.

The extent of this bending is quantified in Figure 8, which shows sequential deflection profiles for the SiCNT. Removed lipids were not included in calculation. The shift from z=−55 Å (black) to z=−25 Å displays the tendency of the membrane to track along with the SiCNT indenter. The green points show that even after the indenter receded beyond its original starting position, the membrane had not yet begun to recover. This is in contrast to the minimal disruption and self-sealing displayed by the CNT in Figure 7 and Table 2.

## 4. Conclusions

We performed SMD simulations of membrane indentation by nanotubes inclined at different angles, speeds, and directions. In all cases, increasing the nanotube’s angle away from the perpendicular and increasing the speed both increased the force required to penetrate the membrane, and the resultant membrane deflection. These effects were most significant for indenters making sidelong contact with the membrane, i.e., obliquely angled but traveling perpendicular to the membrane. It was evident that this mode of indentation led to a qualitative change in the mechanics of penetration from axially directed piercing to more inefficient continuous slicing. Our withdrawal simulations only encompassed the most extreme (15°), but the same trends for speed and indentation mode were observed, albeit reduced.

If we consider the removal of lipids from the membrane to be the ultimate hallmark of permanent damage, then higher indentation and withdrawal rates with motion closely aligned to the nanotube axis should be preferred. These are also the parameters that allow for the most efficient membrane self-sealing. In the context of drug delivery via a nanoneedle, this would mean that care must be taken when affixing the nanotube to the implement. It would also seem that a narrower CNT should be preferred over a SiCNT as the nanotube material. However, the increased impact of an SiCNT may in part be due to the stronger interactions with the DMPC lipids and not solely its size. This effect was not fully explored herein. Many applications involve the functionalization of the surfaces of nanotubes, which could alter this affinity and could be an area for further research.

## Figures and Tables

**Table 1:** Averaged number of lipids removed during indentation.

Angle	CNT or SiCNT	Along Tube Axis	Along *z*-Axis
low rate			
4.5°	CNT	2.9 (1.9)	3.8 (1.5)
9°	CNT	3.1 (1.5)	3.9 (1.4)
15°	CNT	3.9 (2.2)	4.1 (2.4)
15°	SiCNT	7.5 (2.4)	10.0 (3.2)
high rate			
4.5°	CNT	2.4 (1.6)	2.8 (2.2)
9°	CNT	2.6 (1.8)	3.1 (1.4)
15°	CNT	2.7 (2.4)	4.4 (2.2)
15°	SiCNT	4.9 (2.4)	8.0 (2.6)

**Table 2:** Number of lipids removed during withdrawal.

CNT or SiCNT	Along Tube Axis	Along *z*-Axis
low rate		
CNT	1.9 (1.2)	1.2 (1.0)
SiCNT	4.8 (2.0)	4.3 (1.2)
high rate		
CNT	1.0 (0.7)	0.4 (0.5)
SiCNT	1.4 (1.1)	1.9 (0.9)

## Data Availability

Data available directly from the authors.

## Supplementary Materials

The following are available online at https://www.mdpi.com/article/10.3390/s21217011/s1, Figure S1: SMD force vs. indenter depth for 9° simulation runs, Figure S2: Total interaction energy between nanotube atoms and lipid molecules for cases at 15°, Figure S3: Average work input by SMD atoms vs. indenter depth for the indentation process, Figure S4: Total work input by SMD atoms vs. indenter depth for the withdrawal process, Figure S5: The average membrane density profile along the *z*-axis for withdrawal.
