# Supplementary material for: Steered Molecular Dynamics of Lipid Membrane Indentation by Carbon and Silicon-Carbide Nanotubes—The Impact of Indenting Angle Uncertainty
Source: Sensors (Basel). 2021 Oct 22;21(21):7011. doi: 10.3390/s21217011 (PMC8587279; doi:10.3390/s21217011)
Supplement: Supplementary file 1 [file sensors-21-07011-s001.zip › sensors-1407529-supplementary.pdf]

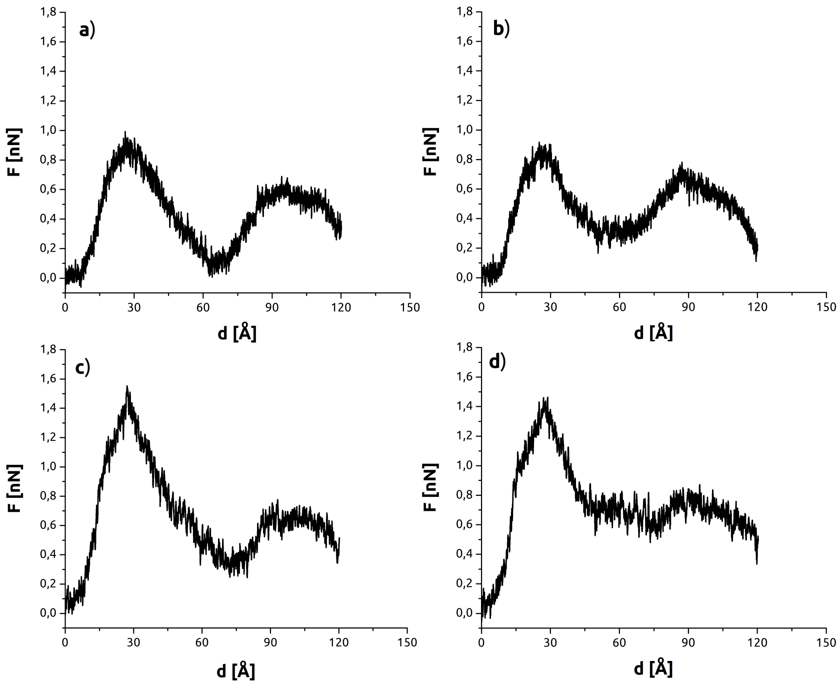

Figure S1. SMD force vs indenter depth for 9° simulation runs. a) Low rate ($v = 0.5$ m/s), tube-axis indentation; b) Low rate ($v = 0.5$ m/s), z-axis indentation; c) High rate ($v = 2.0$ m/s), tube-axis indentation; d) High rate ($v = 2.0$ m/s), z-axis indentation

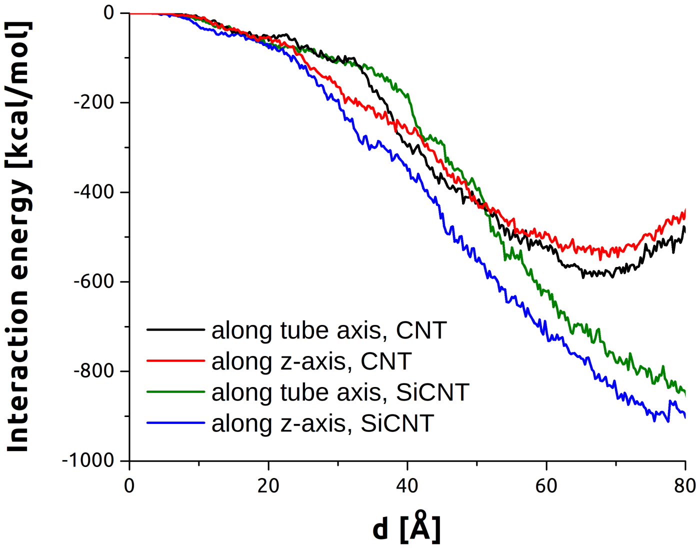

Figure S2. Total interaction energy between nanotube atoms and lipid molecules for cases at 15°. Each curve represents a single run.

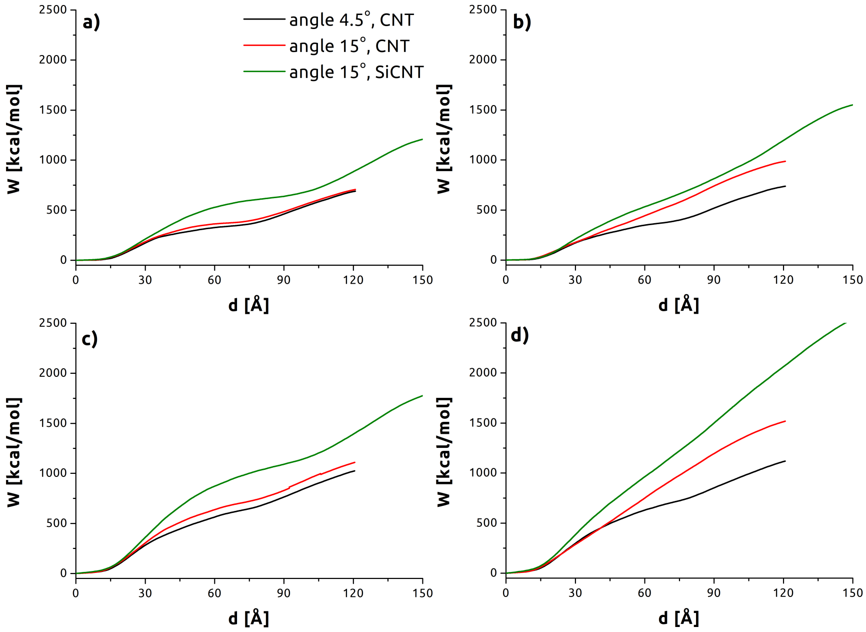

Figure S3. Average work input by SMD atoms vs indenter depth for the indentation process. Curves represent averages over each case. a) Low rate ($v = 0.5$ m/s), tube-axis indentation; b) Low rate ($v = 0.5$ m/s), z-axis indentation; c) High rate ($v = 2.0$ m/s), tube-axis indentation; d) High rate ($v = 2.0$ m/s), z-axis indentation

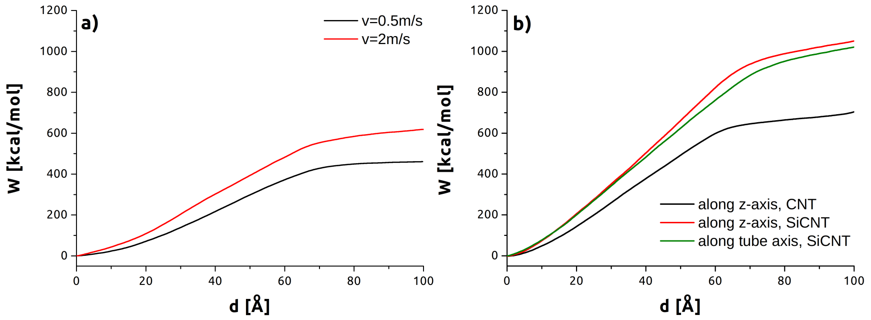

Figure S4. Total work input by SMD atoms vs indenter depth for the withdrawal process. Curves represent averages over each case. All cases simulated were angled at 15°. Left: Rate comparison (CNT, tube-axis indentation). Right: Nanotube comparison (CNT/SiCNT high rate indentation).

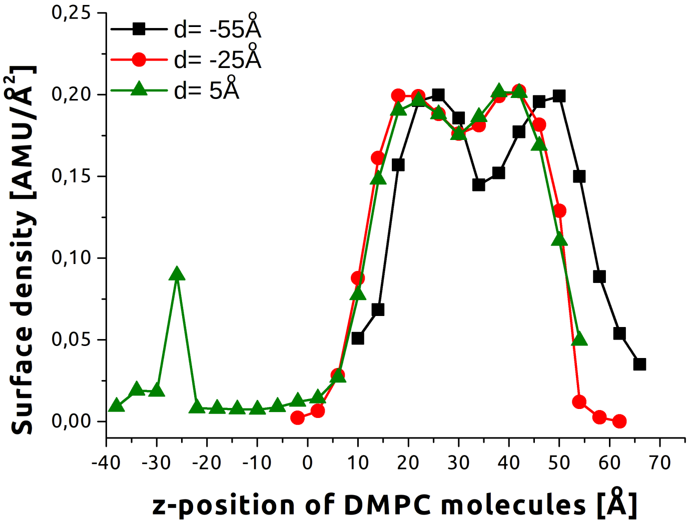

Figure 5. The average membrane density profile along the *z*-axis for withdrawal. Data is for the SiCNT indenter at 2 m/s, a 15° angle directed along the *z*-axis
